# Comparative Validation of Scintillator Materials for X-Ray-Mediated Neuronal Control in the Deep Brain

**DOI:** 10.3390/ijms252111365

**Published:** 2024-10-22

**Authors:** Mercedes Hildebrandt, Masanori Koshimizu, Yasuki Asada, Kansai Fukumitsu, Mahito Ohkuma, Na Sang, Takashi Nakano, Toshiaki Kunikata, Kai Okazaki, Noriaki Kawaguchi, Takayuki Yanagida, Linyuan Lian, Jianbing Zhang, Takayuki Yamashita

**Affiliations:** 1 Department of Physiology, School of Medicine, Fujita Health University, Toyoake 470-1192, Aichi, Japan; 81021041@fujita-hu.ac.jp (M.H.); kansai.fukumitsu@fujita-hu.ac.jp (K.F.); m-ohkuma@fujita-hu.ac.jp (M.O.); n.sang@aichi-cc.jp (N.S.); 2 Research Institute of Electronics, Shizuoka University, Hamamatsu 432-8011, Shizuoka, Japan; koshimizu.masanori@shizuoka.ac.jp; 3 Faculty of Radiological Technology, School of Medical Science, Fujita Health University, Toyoake 470-1192, Aichi, Japan; asada@fujita-hu.ac.jp; 4 International Center for Brain Science, Fujita Health University, Toyoake 470-1192, Aichi, Japan; takashi.nakano@fujita-hu.ac.jp; 5 Department of Computational Biology, School of Medicine, Fujita Health University, Toyoake 470-1192, Aichi, Japan; 6 Division of Materials Science, Nara Institute of Science and Technology (NAIST), Ikoma 630-0192, Nara, Japan; kunikata.toshiaki.kt1@ms.naist.jp (T.K.); okazaki.kai.of0@ms.naist.jp (K.O.); n-kawaguchi@ms.naist.jp (N.K.); t-yanagida@ms.naist.jp (T.Y.); 7 Key Laboratory of Materials Physics of Ministry of Education, School of Physics, Zhengzhou University, Zhengzhou 450052, China; lianlinyuan@zzu.edu.cn; 8 School of Integrated Circuits, Wuhan National Laboratory for Optoelectronics, Huazhong University of Science and Technology, Wuhan 430074, China; jbzhang@hust.edu.cn

**Keywords:** optogenetics, X-rays, scintillator, nanoparticles, toxicity, deep brain stimulation, dopamine, electrophysiology

## Abstract

When exposed to X-rays, scintillators emit visible luminescence. X-ray-mediated optogenetics employs scintillators for remotely activating light-sensitive proteins in biological tissue through X-ray irradiation. This approach offers advantages over traditional optogenetics, allowing for deeper tissue penetration and wireless control. Here, we assessed the short-term safety and efficacy of candidate scintillator materials for neuronal control. Our analyses revealed that lead-free halide scintillators, such as Cs_3_Cu_2_I_5_, exhibited significant cytotoxicity within 24 h and induced neuroinflammatory effects when injected into the mouse brain. In contrast, cerium-doped gadolinium aluminum gallium garnet (Ce:GAGG) nanoparticles showed no detectable cytotoxicity within the same period, and injection into the mouse brain did not lead to observable neuroinflammation over four weeks. Electrophysiological recordings in the cerebral cortex of awake mice showed that X-ray-induced radioluminescence from Ce:GAGG nanoparticles reliably activated 45% of the neuronal population surrounding the implanted particles, a significantly higher activation rate than europium-doped GAGG (Eu:GAGG) microparticles, which activated only 10% of neurons. Furthermore, we established the cell-type specificity of this technique by using Ce:GAGG nanoparticles to selectively stimulate midbrain dopamine neurons. This technique was applied to freely behaving mice, allowing for wireless modulation of place preference behavior mediated by midbrain dopamine neurons. These findings highlight the unique suitability of Ce:GAGG nanoparticles for X-ray-mediated optogenetics. The deep tissue penetration, short-term safety, wireless neuronal control, and cell-type specificity of this system offer exciting possibilities for diverse neuroscience applications and therapeutic interventions.

## 1. Introduction

Recent advances in biomedical technologies employing light-sensitive proteins such as microbial rhodopsins (opsins) have created significant opportunities for manipulating various cellular functions in living animals [1,2,3,4]. Optogenetics, particularly in neuroscience, is one of the most widely applied forms of this approach [1,3,5]. Clinical treatments for various diseases could also benefit from light-mediated methods that enable precise control over well-defined cellular populations [3,6]. Most of these technologies rely on visible light, commonly blue light, to activate the light-sensitive proteins. However, visible light is heavily scattered and absorbed by biological tissues [7,8], making it challenging to manipulate light-sensitive proteins beyond 1 mm of tissue with optogenetics. To address this limitation, researchers often implant rigid optical fibers near target regions, which can cause several issues, including surgical damage to the tissue [9]. Recent studies in neuroscience have reported transcranial stimulation of neurons at depths of several millimeters in mice using opsins with extremely high light sensitivity [10,11,12]. However, subcortical regions in the larger brains of humans or monkeys at a depth of centimeters are essentially inaccessible without optical fiber implants.

To deliver visible light deep within tissue without implanted optical fibers, researchers have explored using upconversion materials [13,14,15,16,17], which emit visible light upon near-infrared (NIR) irradiation [18,19]. NIR light (650–1350 nm) penetrates tissue more deeply than visible light [7,8], and upconversion luminescence emitted from injected nanoparticles can effectively activate opsins to actuate neurons in living animals [16,17]. However, this method has the drawback of causing tissue heating during NIR irradiation [20]. In addition, even NIR light does not well-penetrate more than 1 cm of biological tissue. An alternative approach involves scintillators, which emit visible light upon X-ray absorption. Our previous work [20] demonstrated that micrometer-sized particles of a yellow-emitting inorganic scintillator, cerium-doped gadolinium aluminum gallium garnet (Ce:GAGG) [21,22], can be safely implanted in the mouse brain. We successfully manipulated midbrain dopamine (DA) neurons, as evidenced by c-Fos expression and behavioral tests in mice [20]. While other groups have reported similar concepts with different scintillator materials [23,24], direct in vivo observation of neuronal firing has been lacking, leaving the efficacy of X-ray-mediated optogenetics unclear. Moreover, there has been no direct comparison of different scintillators’ usability.

For use in living animal tissues, scintillators must emit bright radioluminescence, remain stable in solid form, and resist dissolution in extracellular fluid. They should also be biocompatible, non-cytotoxic, and non-inflammatory. Ce:GAGG is an ideal scintillator for these purposes due to its high light yield (46,000 photons/MeV [21,22]), stability in the tissue, ability to be injected as particles, and permanent retention at the injection site [20]. Ce:GAGG is also known to be non-cytotoxic and non-inflammatory [20]. However, the size of Ce:GAGG particles is a concern. Previously, we ground bulk Ce:GAGG crystals into micrometer-sized particles with an average diameter of ~2 µm [20]. Nanometer-sized particles would be ideal for broader applications [25,26], allowing them to be functionalized to target specific proteins or cell types. Therefore, synthesized Ce:GAGG nanoparticles, which have recently been reported [27], would be an attractive candidate for X-ray optogenetics. Other scintillators, such as non-hydrophobic halide scintillators like Cs_3_Cu_2_I_5_ [28] and (C_38_H_34_P_2_)MnBr_4_ [29], which reportedly have higher light yields than Ce:GAGG, are also attractive candidates for X-ray-mediated optogenetics. Recently reported europium-doped GAGG (Eu:GAGG), which exhibits orange radioluminescence (peak emission wavelength: ~580 nm [30]) with a relatively high light yield (36,000 photons/MeV [30]), may also be beneficial for activating red-shifted opsins such as ChRmine [11,31].

In this study, we aimed to identify novel scintillator materials suitable for X-ray-mediated optogenetics. We first tested the short-term safety of the candidate scintillators mentioned above. Although Ce:GAGG microparticles have been shown to be non-cytotoxic [20], achieving high purity in synthesized nanoparticles is challenging, with potential risks of contamination affecting bio-safety. Therefore, we evaluated the short-term safety of Ce:GAGG nanoparticles, as well as other scintillators that have not been previously tested. We then assessed the efficacy of these scintillators in manipulating neuronal activities using in vivo electrophysiological measurements under X-ray irradiation (X-irradiation). We used an X-ray machine designed for human radiography to provide sufficient space for recording under X-irradiation. Our results indicate that halide scintillators exhibit significant toxicity, while Ce:GAGG and Eu:GAGG are tolerated in the short term with no observable signs of neuroinflammation after injection into the mouse brain. Furthermore, by carefully addressing electrical noise from the X-ray machine, we successfully recorded neuronal firings in awake mouse brains upon X-irradiation. We found that synthesized Ce:GAGG nanoparticles can efficiently manipulate the activities of neurons surrounding the injected particles, achieving cell-type specificity and inducing behavioral changes.

## 2. Results

### 2.1. Short-Term Safety of Candidate Scintillators

**Figure 1:**
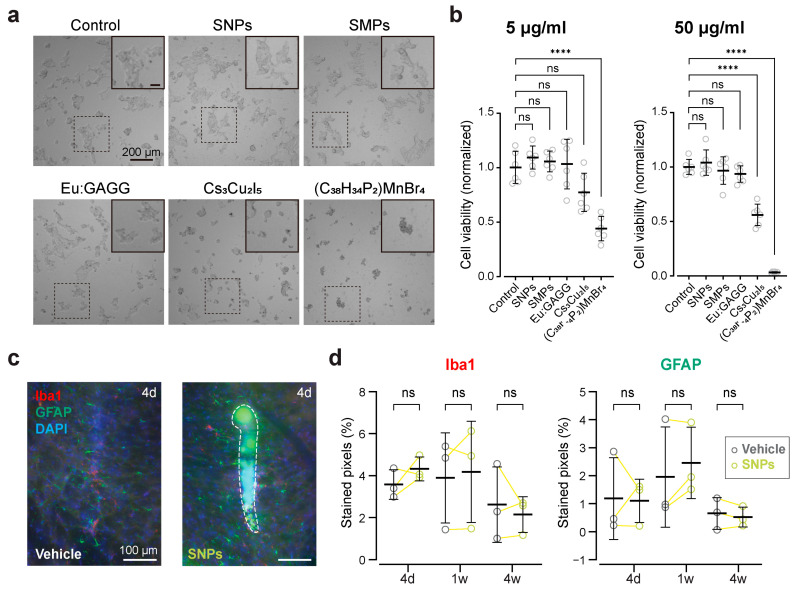
Short-term safety assessment of scintillators for potential use in X-ray-mediated optogenetics. (**a**) Representative images of cultured HEK293 cells incubated for 24 h with various scintillator particles at a concentration of 50 μg/mL. Control cells were not exposed to any scintillator particles. Insets show magnified views of the area indicated by dotted squares. Scale bar = 200 (main image) and 50 μm (insets). (**b**) Cell viability measured 24 h post-incubation with scintillator particles at 5 μg/mL (left) and 50 μg/mL (right). Values are normalized to the mean of the control group (*n* = 6 for each). **** *p* < 0.0001; ns, not significant; Dunnett’s multiple comparison test versus control. (**c**) Epi-fluorescence images of coronal brain slices showing immunostaining for microglia (Iba1, red) and astrocytes (GFAP, green) at the injection sites of either vehicle (left) or SNPs (right, outlined with a dashed line). We injected both vehicle and SNPs into different hemispheres of the same mouse, with slices collected 4 days (4 d), 1 week (1 w), or 4 weeks (4 w) post-injection. Blue: DAPI. (**d**) Semi-quantification of Iba1 (left) and GFAP (right) immunoreactivity expressed as the percentage of stained pixels in a 100 μm × 100 μm square near the injection traces (*n* = 3 for each). ns, not significant; paired *t*-tests with Bonferroni’s correction for multiple comparisons. Open circles represent individual data points, and yellow lines denote individual mice. Black horizontal lines and error bars indicate mean ± SD. Statistical details are shown in Table S1.

We first examined the cytotoxicity of candidate scintillators in vitro prior to conducting in vivo experiments. We chose to use cultured HEK293 cells because they are a well-established model for initial cytotoxicity screening [20]. We tested Ce:GAGG nanoparticles (named as SNPs [27]), Eu:GAGG particles [30], Cs_3_Cu_2_I_5_ nanocrystals [28], and (C_38_H_34_P_2_)MnBr_4_ particles [29]. We also used the biocompatible Ce:GAGG microparticles (named as SMPs [20]) as a negative control. Among these, (C_38_H_34_P_2_)MnBr_4_ particles at 50 μg/mL exhibited pronounced cytotoxicity, nearly abolishing cell viability within 24 h (Figure 1a,b; cell viability: 3.30 ± 0.65%, *n* = 6). In contrast, incubation with SMPs, SNPs, and Eu:GAGG particles did not significantly affect the cell viability compared to the control group without scintillator exposure (Figure 1a,b). Cs_3_Cu_2_I_5_ nanocrystals displayed a dose-dependent cytotoxicity, affecting cell viability at 50 μg/mL but not at 5 μg/mL (Figure 1a,b). To further assess bio-safety, we conducted in vivo experiments by injecting them into mouse brains. SNPs (50 mg/mL, 600 nL) did not induce significant accumulation of astrocytes or microglia at 4 days, 1 week, or 4 weeks post-injection, suggesting no neuroinflammation in the short period (Figure 1c,d). However, injection of Cs_3_Cu_2_I_5_ (50 mg/mL, 200 nL) into the mouse brain caused a severe neuroinflammatory response 4 days post-injection (Figure S1a). Notably, mice injected with (C_38_H_34_P_2_)MnBr_4_ particles (50 mg/mL, 200 nL) did not survive beyond 1 h (*n* = 4 mice). Injection of Eu:GAGG particles (50 mg/mL, 600 nL) resulted in no detectable neuroinflammation after 4 days (Figure S1b). These findings are consistent with our in vitro results, suggesting that SNPs and Eu:GAGG particles are well tolerated in the short term. In contrast, Cs_3_Cu_2_I_5_ nanocrystals may have cytotoxic and neuroinflammatory effects at higher concentrations, and (C_38_H_34_P_2_)MnBr_4_ particles show significant toxicity.

### 2.2. Electrophysiological Assessment of Neuronal Control by X-Ray-Mediated Optogenetics

**Figure 2:**
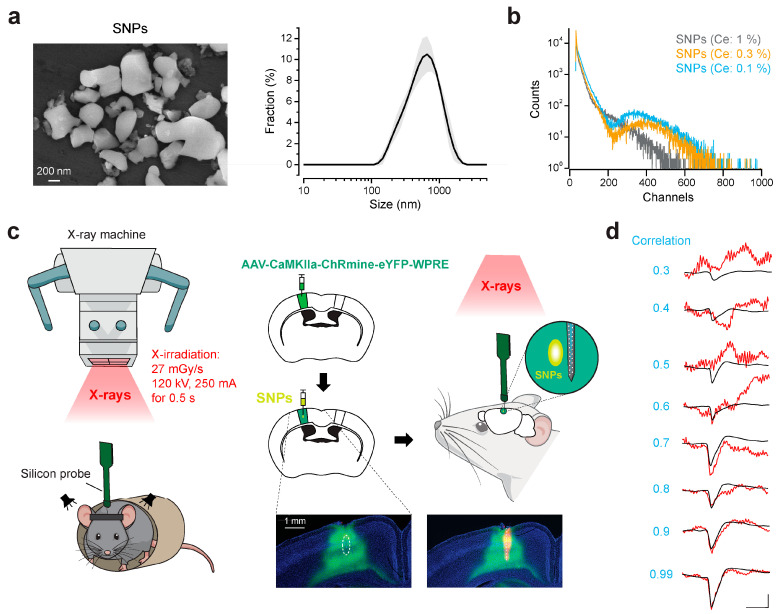
SNPs and their use for X-ray-mediated optogenetics. (**a**) Left, a scanning electron micrograph of SNPs. Right, size distribution of SNPs measured by dynamic light scattering. Mean = 498 ± 8 nm (*n* = 3). The black line and shadow indicate mean ± SD. (**b**) Pulse-height spectra of SNPs synthesized with different Ce concentrations (in mol%). (**c**) Schematic drawing of electrophysiological recordings underneath an X-ray machine designed for human radiography. Note that the electrodes of the silicon probe face injected SNPs. Insets: Epi-fluorescence images of SNPs in S1 region (left, circled) and silicon probe recording trace posterior to SNPs (right, dashed line). Green: ChRmine-eYFP; Red: DiI; Blue: DAPI. (**d**) Average AP waveforms during X-irradiation (red) superimposed with average AP waveforms at other timings (black) and their cross-correlation coefficients (blue). Scale bar: 1 ms, 0.5 mV.

Next, we examined the efficacy of neuronal control with X-ray-mediated optogenetics using SNPs. SNPs had an average diameter of 498 nm (Figure 2a), which is considerably smaller than SMPs [20]. The peak of the emission spectrum of radioluminescence of SNPs is at ~560 nm [27]. Among opsins used for optogenetics, ChRmine is the best suited for photo- or radio-luminescence emitted from Ce:GAGG [20]. The light yield of SNPs (Ce concentration: 0.3 mol%) was measured as 13,800 photons/MeV (Figure 2b). We introduced the expression of ChRmine in neurons in the primary somatosensory cortex (S1) of mice by injecting an adeno-associated virus (AAV) vector (AAV9-CaMKII-ChRmine-eYFP). ChRmine is tagged with enhanced yellow fluorescent protein (eYFP), allowing us to easily verify its expression through green fluorescence (Figure 2c). After ≥11 days of AAV injection, we injected SNPs (50 mg/mL in Ringer’s solution, 600 nL) at the AAV injection site. Using a silicon neural probe, we performed electrophysiological recordings of neuronal firings from awake head-fixed mice under an X-ray machine 6–17 days after injection of SNPs (Figure 2c). The exact timelines of AAV/SNP injections for individual mice are shown in Table S2. We inserted the probe so that the electrodes face the SNP injection site at a distance of a few hundred micrometers. We used an X-ray machine for human radiography and irradiated X-rays for 0.5 s at a dose rate of 27 mGy/s (tube voltage: 120 kV, tube current: 250 mA). We have noticed that some of the recordings were significantly contaminated with electrical noises during X-irradiation. We therefore analyzed action potential (AP) waveforms obtained from individual single units (putative neurons) and compared the average AP waveforms during X-irradiation and those at other timings (Figure 2d). To analyze neuronal firings stringently, we excluded from analysis the data with a cross-correlation coefficient between these waveforms less than 0.85 [32].

**Figure 3:**
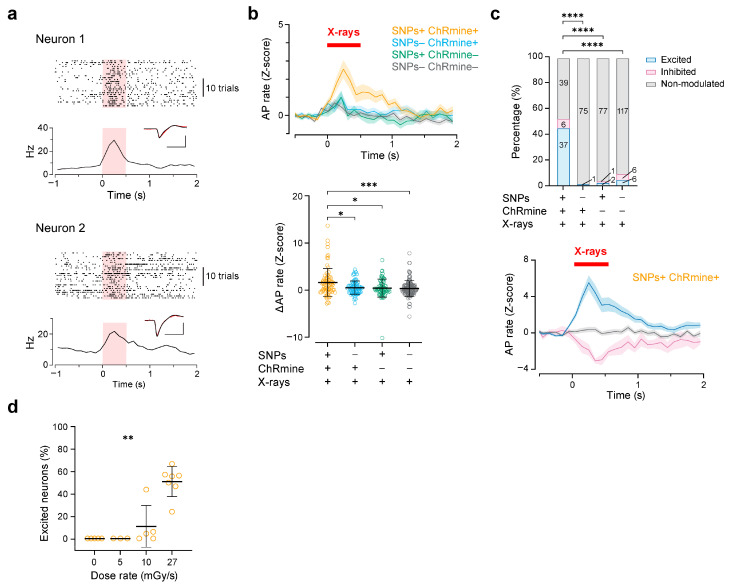
X-ray-mediated optogenetics using SNPs caused excitation of the local neuronal circuit. (**a**) Raster plots and peri-stimulus time histograms (PSTHs) of two representative neurons excited by X-irradiation (red shadow). Inset: average AP waveforms of these neurons during X-irradiation (red) and other timings (black, superimposed). The cross-correlation coefficient between these waveforms was 0.963 for neuron 1 and 0.950 for neuron 2. Scale bar: 1 ms, 0.5 mV. (**b**) Top: grand average of Z-scored PSTHs of different conditions (SNPs+ ChRmine+ [orange]: *n* = 82 neurons from 5 mice; SNPs– ChRmine+ [cyan]: *n* = 76 neurons from 6 mice; SNPs+ ChRmine– [green]: *n* = 80 neurons from 2 mice; SNPs– ChRmine– [gray]: *n* = 129 neurons from 3 mice). Bottom: Z-scored mean AP rate changes induced by X-irradiation. SNPs– ChRmine+, * *p* = 0.0458; SNPs+ ChRmine–, * *p* = 0.0169; SNPs– ChRmine–, *** *p* = 0.0007; Dunn’s post hoc tests vs. SNPs+ ChRmine+ group. (**c**) Top: proportions of the neurons that were excited (blue), inhibited (magenta), and non-modulated (gray) by X-irradiation. **** *p* < 0.0001, Chi-square tests vs. SNPs+ ChRmine+ group with Bonferroni’s correction for multiple comparisons. Bottom: grand average Z-scored PSTHs of these neurons in the SNPs+ ChRmine+ group. (**d**) Pooled data of the proportion of the neurons excited by X-irradiation with varying dose rates (0 mGy/s, *n* = 5 recordings from 4 mice; 5 mGy/s, *n* = 3 recordings from 2 mice; 10 mGy/s, *n* = 5 recordings from 4 mice; 27 mGy/s, *n* = 7 recordings from 5 mice). ** *p* = 0.0012, Kruskal–Wallis test. Colored lines and shadows in the PSTHs denote mean ± SEM. Open circles represent individual data points. Black horizontal bars and error bars indicate mean ± SD. Statistical details are shown in Table S1.

Next, we tested whether the firing rates of neurons significantly changed during X-irradiation. The average firing rate of the neuronal population in the mice where AAV and SNPs were injected was significantly increased by X-irradiation (dose rate: 27 mGy/sduration: 0.5 s) compared to that of control groups without injection of either AAV or Ce:GAGG, or both of them (Figure 3a–c). We found that 45% (37 out of 82) of the neurons increased their firing rate upon X-irradiation, while 7% (6 out of 82) decreased their firing rate, and 48% (39 out of 82) showed no change in their firing rate upon X-irradiation (Figure 3c). In the neurons excited by X-irradiation, their mean firing rate peaked at around 250 ms after the X-irradiation onset (Figure 3c). Moreover, the proportion of excited neurons was increased depending on the dose rate of X-irradiation (Figure 3d). In contrast, the proportion of excited neurons in control conditions was significantly smaller (Figure 3c; SNPs–ChRmine+: 1%, 1 out of 76 neurons; SNPs+ ChRmine–: 2.5%, 2 out of 80 neurons; SNPs–ChRmine–: 5%, 6 out of 129 neurons). These results suggest that radioluminescence emitted from SNPs in vivo activated ChRmine-expressing neurons and the neuronal circuits they are embedded in.

**Figure 4:**
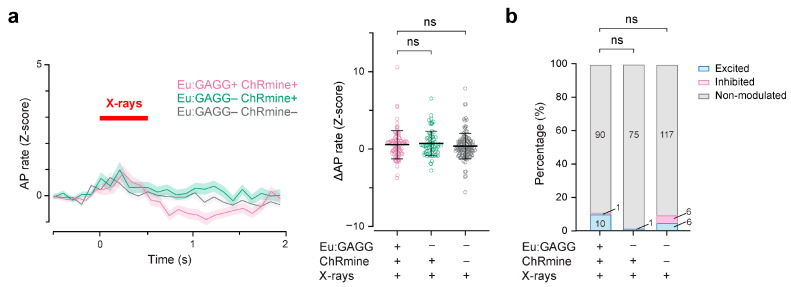
X-ray-mediated optogenetics using Eu:GAGG particles did not induce discernible neuronal dynamics. (**a**) Left, grand average of Z-scored AP rate of all neurons of test group (Eu:GAGG+ ChRmine+, magenta) and two control groups (Eu:GAGG– ChRmine+, green; Eu:GAGG–ChRmine–, gray). Right, Z-scored AP rate changes induced by X-irradiation. ns, not significant, Dunn’s post hoc tests. (**b**) Proportions of the neurons that were excited (blue), inhibited (magenta), and non-modulated (gray) by X-irradiation. ns, not significant, Chi-square tests with Bonferroni’s correction for multiple comparisons. Colored lines and shadows in the PSTHs denote mean ± SEM. Open circles represent individual data points. Black horizontal bars and error bars indicate mean ± SD. Statistical details are shown in Table S1.

We further tested the efficacy of X-ray-mediated optogenetics using Eu:GAGG particles. The emission spectrum of Eu:GAGG radioluminescence with a peak of ~580 nm [30] is close to the optimal activation wavelength of ChRmine [31]. Therefore, we used ChRmine for opsins for the test. We virally introduced ChRmine expression in the S1 cortex of mice and injected Eu:GAGG particles at the same site, as we did to test SNPs (Figure 2c, Table S2). We then recorded neuronal firings with a silicon probe. With including data with high correlation of AP waveforms during stimulus and at other timings (correlation coefficient, ≥0.85), we found that there was no significant difference in mean AP firing rates during X-irradiation between the test group (Eu:GAGG + ChRmine+) and other controls (Eu:GAGG– ChRmine+ and Eu:GAGG– ChRmine–, same data as control groups for SNP experiments [Figure 3]) (Figure 4a). In the test group, 10% (10 out of 101) of the neurons increased their firing rate upon X-irradiation. The proportion of excited and inhibited neurons in the test group was not significantly different from those of control groups (Figure 4b). These results indicated that radioluminescence from injected Eu:GAGG particles did not sufficiently activate ChRmine-expressing neurons in vivo.

### 2.3. X-Ray-Mediated Optogenetics of Midbrain Dopamine Neurons Using SNPs

**Figure 5:**
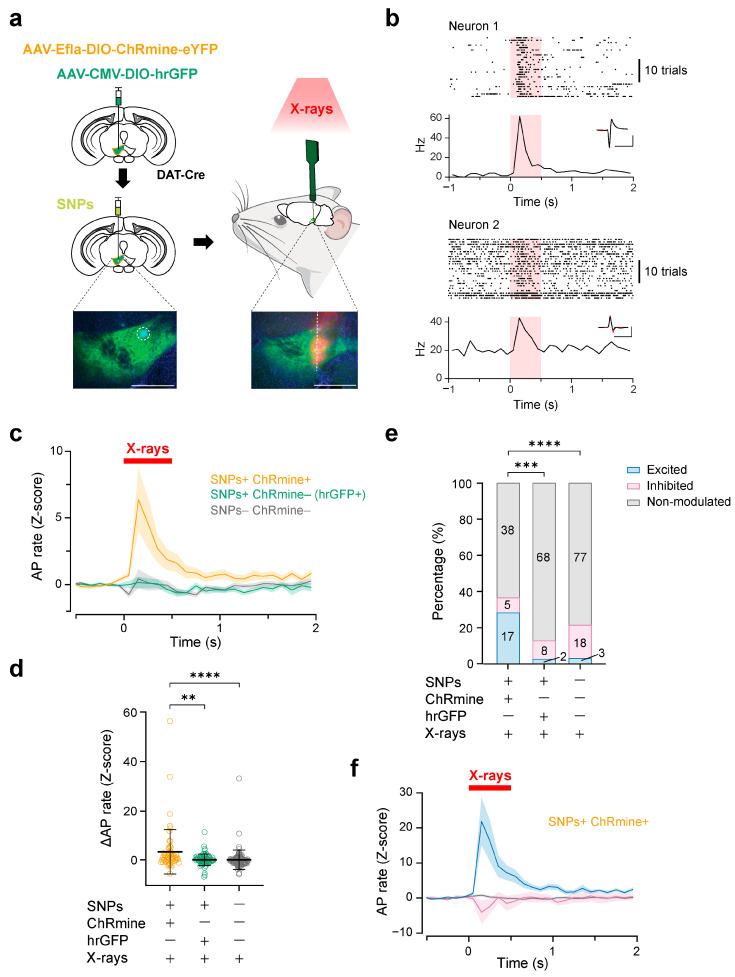
Deep brain stimulation of VTA-DA neurons via X-ray-mediated optogenetics. (**a**) Schematic of the experiment. Insets: epi-fluorescence images of SNPs in the VTA (left, dashed white circle) and silicon probe trace posterior to SNPs (right, dashed white line). The electrodes on the silicon probe faced the SNP-injection site. Green: ChRmine-eYFP; Red: DiI; Blue: DAPI. Scale bars: 500 µm. (**b**) Raster plots and PSTHs of two representative neurons excited by X-irradiation (red shadow). Inset: average AP waveform of these neurons during X-irradiation (red) and other timings (black, superimposed). The cross-correlation coefficient between these waveforms was 0.998 for neuron 1 and 0.966 for neuron 2. Scale bar: 1 ms, 0.5 mV. (**c**) Grand average of Z-scored AP rate of all neurons of test group (SNPs+ ChRmine+, orange) and two control groups (SNPs + ChRmine – [hrGFP+], green; SNPs – ChRmine-, gray). (**d**) Z-scored AP rate changes induced by X-irradiation. ** *p* = 0.0070, **** *p* < 0.0001, Dunn’s post hoc tests vs. SNPs+ ChRmine+ group. (**e**) Proportions of the neurons that were excited (blue), inhibited (magenta), and non-modulated (gray) by X-irradiation. *** *p* = 0.000225, **** *p* < 0.0001, Chi-square tests with Bonferroni’s correction for multiple comparisons. (**f**) Grand average Z-scored PSTHs of these neurons in the SNPs+ ChRmine+ group. Colored lines and shadows in the PSTHs denote mean ± SEM. Open circles represent individual data points. Black horizontal bars and error bars indicate mean ± SD. Statistical details are shown in Table S1.

Next, we tested whether X-ray-mediated optogenetics using SNPs can achieve cell-type-specific activation of deep brain neurons. We targeted DA neurons in the ventral tegmental area (VTA) of the mouse midbrain, located at a depth of around 4.0–4.6 mm from the brain surface. In the VTA, DA neurons are intermingled with GABAergic and glutamatergic neurons, comprising 55–65% of the total neuronal population [33,34,35]. We induced the expression of ChRmine in the VTA-DA neurons via local injection of AAV vectors (AAV9-EF1a-DIO-ChRmine) in DAT-IRES-Cre mice. With the Cre-Lox recombination, the expression of ChRmine will be restricted in DA neurons (specificity: 98% [20]). We further injected SNPs at the same location as AAV injection and recorded neuronal firings from the VTA of the head-fixed mice (Figure 5a, Table S2). In our recordings from five mice with ChRmine expression, X-irradiation (27 mGy/s, 0.5 s) caused an instant increase in the mean firing rates of recorded neurons (Figure 5b–d). Analysis of individual neurons revealed that 28% (17 out of 60) of the neurons increased their AP rate upon X-irradiation, while 8% (5 out of 60 neurons) decreased their AP rate and 63% (38 out of 60 neurons) showed no significant change in their AP rate upon X-irradiation (Figure 5e,f). As control experiments, we virally introduced hrGFP expressions in the VTA-DA neurons instead of ChRmine and injected SNPs in the VTA (Figure S2), or we injected neither AAVs nor SNPs, and performed silicon probe recordings from the VTA. In these cases, there was no change in the mean firing rates (Figure 5c,d). Moreover, a significantly smaller fraction of neurons increased their AP rate upon X-irradiation compared to the test group with ChRmine expression (Figure 5e). These results suggest the adequate stimulation of VTA-DA neurons by X-ray-mediated optogenetics.

**Figure 6:**
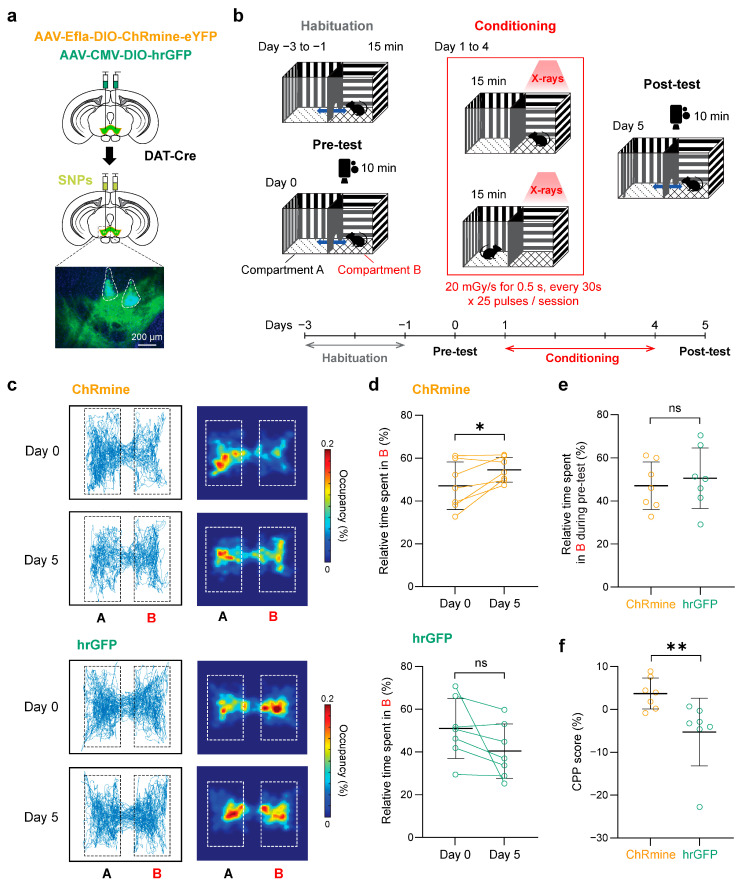
X-ray-mediated optogenetics using SNPs applied to behavioral tests with freely moving mice. (**a**) Schematic of the experiment. Inset: epi-fluorescence image of a coronal section containing the left VTA with SNPs (dashed outline) injected into two locations. Green: ChRmine-eYFP; blue: DAPI. (**b**) Schematic of CPP tests. (**c**) Representative tracking data (left) and corresponding heat maps (right) for ChRmine− (top) and hrGFP− (bottom) expressing mice. Dotted rectangles show the area used for analysis. (**d**) Relative time spent in the X-irradiated compartment of ChRmine (top, 7 mice) and hrGFP (bottom, 7 mice) mice in the pre-test (day 0) compared to that in the post-test (day 5). * *p* = 0.0353; ns, not significant; paired *t*-test. (**e**) Quantification of initial preference for the X-irradiated compartment. ns, not significant; unpaired *t*-test. (**f**) Quantification of CPP scores. ** *p* = 0.0041; Mann–Whitney *U* test. Open circles represent individual data points, and the lines connecting the open circles denote individual mice. Black horizontal bars and error bars indicate mean ± SD. Statistical details are shown in Table S1.

Behavioral conditioning with transient activation of VTA-DA neurons is sufficient to modulate place preference behaviors [36,37]. We therefore tested whether the activation of VTA-DA neurons with X-ray-mediated optogenetics using SNPs could affect place preference behaviors. We induced the expression of ChRmine or hrGFP in VTA-DA neurons through bilateral viral injections and injected SNPs into the VTA of both hemispheres (Figure 6a, Table S2). The conditioned place preference (CPP) test was performed by placing the mice into a test chamber with two compartments, only one of which was irradiated with 0.5 s X-ray pulses at the dose rate of 20 mGy/s (every 30 s, 25 times/day) (Figure 6b and Figure S3). The initial place preference was not different between the ChRmine-expressing and hrGFP-expressing mice (Figure 6c–e). However, after four days of conditioning, mice expressing ChRmine exhibited a significantly higher preference for the X-ray-conditioned compartment, while hrGFP-expressing control mice showed no change in preference (Figure 6c–e). The CPP score was significantly higher in ChRmine-expressing mice compared to control mice (Figure 6f). These results suggest that X-ray-mediated remote optogenetics using SNPs can be effectively applied for cell-type-specific targeting in deep brain regions during free-moving behavioral experiments in mice.

## 3. Discussion

X-ray-mediated optogenetics is an emerging technology that leverages X-rays to remotely induce radioluminescence in scintillator particles within living tissue, ultimately activating light-sensitive proteins in surrounding cells to manipulate cellular functions [9]. The feasibility of this technique has been demonstrated in neuroscience studies, where X-ray-induced scintillation has been employed to actuate opsin-expressing neurons [20,23,24]. In this study, we evaluated the short-term safety and efficacy of candidate scintillators to enhance the application of X-ray-mediated optogenetics. Overall, our findings demonstrate that X-ray-mediated remote optogenetics using Ce:GAGG nanoparticles (SNPs) can achieve precise, cell-type-specific deep brain stimulation. This not only validates the utility of SNPs in neuronal manipulations but also highlights their potential to revolutionize neuromodulation strategies in both fundamental neuroscience research and future clinical applications.

The application of smaller scintillator particles poses several challenges, particularly concerning their physical properties [38,39]. Smaller particles have a significantly higher surface-to-volume ratio compared to bulk crystals, leading to increased surface defects and non-radiative recombination sites. These defects can trap excited electrons and holes, hindering efficient recombination and subsequent light emission, thereby reducing the overall scintillation yield. Additionally, the shorter energy migration pathways within smaller particles are more vulnerable to disruption by surface states or other imperfections. Our measurements confirmed these issues, demonstrating a notable reduction in scintillation light yield for SNPs, underscoring the inherent trade-offs between particle size and efficiency that must be carefully balanced when designing scintillators for biomedical applications.

Nevertheless, our study showed that SNPs, despite these challenges, have the potential to be effective in X-ray-mediated optogenetics. After confirming the short-term safety of SNPs (Figure 1), we found that their radioluminescence was sufficient to activate ChRmine-expressing neurons, effectively stimulating local neuronal circuits (Figure 2 and Figure 3). Moreover, SNPs enabled cell-type-specific activation of neurons in deep brain regions (Figure 5), offering significant advantages for wireless behavioral experiments (Figure 6). In our cortical recordings, X-irradiation excited 45% of the neurons, while in the VTA recordings, 28% of neurons were activated. This discrepancy is likely due to the specificity of ChRmine expression in the VTA, where it is expressed exclusively in DA neurons, which constitute only 55–65% of the population. When considering the proportion of DA neurons, X-ray-mediated optogenetics activated 43–51% of DA neurons, a value comparable to our cortical data. These findings are in line with those from transcranial red laser stimulation studies, which have reported activation of around 30% of VTA neurons [11]. However, due to the superior tissue penetration capacity of X-rays, X-ray-mediated optogenetics presents a more versatile and effective option for manipulating deep brain regions, surpassing the limitations of transcranial light stimulation. This advantage positions X-ray-mediated optogenetics as a potentially transformative tool in the investigation and treatment of deep-seated neural disorders.

Ce:GAGG crystals and their particles are known to be stable and resistant to degradation in the brain [20]. In this study, SNPs apparently remained stable around the injection site (Figure S2) and effectively activated neurons for over weeks post-injection. This suggests that, like SMPs [20], SNPs may become permanently embedded at the injection site. We employed light-sensitive ion channels (ChRmine) expressed on the plasma membrane as target molecules for activation by radioluminescence emitted from SNPs. Therefore, the proximity of SNPs to neurons is likely sufficient for activation without requiring internalization. However, if the targeted light-sensitive molecules are intracellular, such as transcription factors, internalization of the nanoparticles may be necessary.

Although we did not observe any discernible neuroinflammatory effects following SNP injections (Figure 1c,d), consistent with our in vitro cytotoxicity data, we cannot entirely rule out the possibility of tissue damage from the injection procedure or the long-term presence of SNPs in the brain. Further investigation, including long-term biocompatibility tests in neuronal cell cultures and the use of a TUNEL assay to assess apoptotic cell death, will provide important insights into the stability and safety of SNPs over extended periods. These studies will be essential for fully evaluating the biocompatibility of SNPs and their potential for clinical applications.

Even in control conditions, a minor fraction of neurons exhibited significant changes in firing rate during X-irradiation (Figure 3 and Figure 5). The X-ray machine used in this study generates sound during irradiation, and it is known that such instantaneous noise can induce transient orofacial movements, potentially affecting global neuronal activity [40,41,42,43]. Although we habituated the mice to loud white noise and conducted the experiments under these conditions, these factors may have influenced our recordings. Future studies should aim to mitigate such confounding factors by improving the experimental setup.

In addition to evaluating SNPs, we also examined lead-free halide scintillators, including Cs_3_Cu_2_I_5_ and (C_38_H_34_P_2_)MnBr_4_, which have garnered attention due to their exceptionally high light yields and the potential to be prepared as hydrophobic particles [9]. These materials were developed to circumvent the toxicity concerns associated with lead (Pb), which prompted us to evaluate their applicability in X-ray-mediated optogenetics. However, our studies revealed significant biocompatibility challenges with these materials. (C_38_H_34_P_2_)MnBr_4_ particles exhibited pronounced cytotoxicity in vitro, corroborated by in vivo experiments demonstrating immediate lethality in mice after injecting less than 50 µg of these particles. Cs_3_Cu_2_I_5_ nanocrystals also displayed cytotoxicity in vitro, and injecting Cs_3_Cu_2_I_5_ nanocrystals into the mouse brain caused evident neuroinflammation centered at the injection site. The threshold concentration at which toxicity occurs and the precise mechanisms underlying their cytotoxic or inflammatory effects remain unclear. Surface modification with silica or polyethylene glycols may offer a strategy to mitigate their toxicity [16,44,45,46]. Further research into these mitigation strategies will be essential for advancing the safe application of these promising scintillators in neurobiological studies.

Apart from the toxicity associated with the scintillator particles, X-ray toxicity is another critical concern in animal experiments. A thorough examination of this issue revealed that a cumulative radiation dose of ≤1 Gy does not produce detrimental effects on radiosensitive cells in the mouse body [20]. Our behavioral experiments were conducted with a total radiation exposure of 1 Gy. This dosage was carefully selected to balance the need for effective neuronal activation with the imperative to minimize harm to the animals. Given that X-ray-mediated optogenetics is still a developing field, ongoing evaluation of both short- and long-term effects of radiation on animal health is essential. As this technique progresses toward potential therapeutic applications, ensuring the safety of both the scintillators and the X-ray exposure will be paramount. Addressing potential long-term risks and limitations of toxicity in humans is also an important consideration for future applications.

X-ray-mediated optogenetics has promising implications for certain pathologies. Neurological and psychiatric conditions characterized by dysregulated neuronal circuitry, such as depression, anxiety disorders, and some forms of chronic pain, could potentially benefit from this approach. However, several challenges need to be addressed for clinical applications. The use of an X-ray machine poses limitations for continuous stimulation, as required in deep brain stimulation for motor disorders. Enhancing the efficacy of X-ray-mediated optogenetics could induce plastic changes in neuronal circuits, potentially reducing the need for continuous stimulation. Moreover, the current procedure with two separate injections—one for viral vectors and another for scintillator particles—may be suboptimal. The long-term retention of SNPs at the injection site suggests that a single combined injection may also work. Future systematic investigations would be necessary for more accessible and efficient application of this technology. Despite these constraints, the potential to achieve deep tissue stimulation and wireless control may open new therapeutic avenues, particularly for conditions where conventional optogenetic methods are restricted by limited tissue penetration and invasiveness. While this study primarily focuses on advancing X-ray-mediated optogenetics, other forms of energy delivery for deep brain stimulation using ultrasound [47,48,49] and longer wavelength NIR (NIR-II) [50] have also been explored. Continued innovation in this field holds the promise of unlocking new frontiers in the treatment of neurological disorders.

## 4. Materials and Methods

### 4.1. Scintillator Preparations

SNPs were synthesized following the procedure described in a previous paper [27]. The Ce:Gd molar ratio was 0.3:99.7 unless otherwise noted. Metal nitrates and tartaric acid were used as raw materials. Tartaric acid was dissolved in distilled water at a concentration of 0.6 M. The metal nitrates were dissolved in the solution at the stoichiometric ratio, with a total metal concentration of 0.3 M. The solution was stirred at room temperature (RT) for 24 h and then at 80 °C for 2 h. The solution was dried overnight at a temperature above 80 °C to obtain a dry gel, which was subsequently calcined at 1300 °C for 6 h to produce the nanoparticles. SMPs were fabricated from bulk Ce:GAGG crystals synthesized using the conventional Czochralski method as described previously [20].

To synthesize Eu-doped GAGG, a bulk single crystal was first prepared using the floating zone (FZ) method. A mixture of Gd_2_O_3_ (4 N; Furuuchi Chemical, Tokyo, Japan), α-Al_2_O_3_ (4 N; Kojundo Chemical Laboratory, Sakado, Japan), β-Ga_2_O_3_ (4 N; Furuuchi Chemical), and Eu_2_O_3_ (4 N; Furuuchi Chemical) powders was used and combined in stoichiometric mole ratios of (Eu_0.1_, Gd_0.9_)_3_Al_2_Ga_3_O_12_. The mixed powder was formed into a rod using hydrostatic pressure, and the pressed rod was sintered at 1400 °C for 10 h. After sintering, the rod was placed in the FZ furnace for crystal growth at a pulling rate of 3 mm/h. Upon completion of the crystal growth, the single crystal rod was crushed into powder.

The synthesis of Cs_3_Cu_2_I_5_ nanocrystals was carried out using the previously reported method [28]. In a typical synthesis of Cs_3_Cu_2_I_5_ nanocrystals, CuI (0.8 mmol), InI_3_ (0.4 mmol), l-octadecene (10 mL), oleic acid (0.8 mL), and oleylamine (0.8 mL) were mixed in a three-neck flask and degassed for 1 h at 120 °C under vacuum and heated to 180 °C under N_2_, and Cs-oleate solution (2 mL, 0.4 M) was quickly injected; 20 s later, the reaction mixture was cooled using an ice-water bath. Addition of ethyl acetate to the crude solution (2:1 by volume) was followed by centrifugation at 8000 rpm for 3 min, and the supernatant was discarded. The precipitate was dispersed in 5 mL of hexane. After the second centrifugation at 8000 rpm for 3 min, the supernatant was collected. Cs_3_Cu_2_I_5_ nanocrystals were then obtained with hexane evaporation.

The powders of (C_38_H_34_P_2_)MnBr_4_ were obtained by crushing a bulk single crystalline compound. Some pieces of (C_38_H_34_P_2_)MnBr_4_ bulk single crystals were fabricated via the solvent diffusion method. First, C_38_H_34_Br_2_P_2_ (Fujifilm Wako Pure Chemical Corporation, Osaka, Japan, 95.0%) and MnBr_2_ (Fujifilm Wako Pure Chemical Corporation, 97–99.9%) powders in a stoichiometric ratio of 1:1 were dissolved and reacted in a CH_2_Cl_2_ solvent (Fujifilm Wako Pure Chemical Corporation, 99.5%). The solution was stirred for 3 h at RT using a magnetic stirrer at 300 rpm then filtered to obtain (C_38_H_34_P_2_)MnBr_4_ powders. The powders were then dissolved in N,N-dimethylformamide solvent (Fujifilm Wako Pure Chemical Corporation, 99.5%) in a screw-top bottle. The bottle was placed at the bottom of a beaker filled with C_2_H_5_OC_2_H_5_ solvent (Fujifilm Wako Pure Chemical Corporation, 99.5%), which is a poor solvent. Finally, the beaker was covered with aluminum foil and placed in a desiccator at 30 °C. Single crystals, typically a few mm^3^ in size, precipitated after 2–5 days.

To prepare SNPs for injection into the mouse brain, the SNPs were collected in ethanol and ground using an agate mortar to achieve a finer particle size. The ethanol solution carrying the particles was sonicated for 10 min and afterward underwent centrifugation at 20,630× *g* for 30 min to eliminate impurities. The resulting supernatant was discarded. The precipitate containing SNPs was resolved in ethanol. The process of grinding, sonication, centrifugation, and supernatant removal was repeated three times. After evaporation of the remaining solvent, SNPs were dispersed in Ringer’s solution (50 mg/mL) containing the following concentrations (in mM): 135 NaCl, 5 KCl, 5 HEPES, 1.8 CaCl2, 1 MgCl_2_ (adjusted to pH 7.3 with NaOH). The preparation of Eu:GAGG and (C_38_H_34_P_2_)MnBr_4_ particles for experiments followed a similar procedure. The particles were collected in ethanol, ground, and sonicated for 10 min. The supernatant containing smaller particles was centrifuged at 15.871× *g* for 30 s. The resulting precipitate was resuspended in ethanol, and the process was repeated three times. For the application of Cs_3_Cu_2_I_5_, the nanocrystals were collected in Ringer’s solution (50 mg/mL for injection) or culture medium (0.1 mg/mL for in vitro cell experiments) and sonicated for 4 h to improve solubility.

### 4.2. Plasmids

The plasmids pAAV-CaMKIIa-DIO-ChRmine-eYFP and pAAV-Ef1a-DIO-ChRmine-eYFP-WPRE were gifted by K. Deisseroth (Stanford University, Stanford, CA, USA). The plasmid pAAV-CMV-DIO-hrGFP was gifted by D. Ono (Nagoya University, Nagoya, Japan).

### 4.3. In Vitro Cytotoxicity Test

HEK293 cells were seeded into 96-well plates at a density of 10^4^ cells per well. One day later, solutions (10 or 100 μg/mL in culture medium) of each scintillator (Ce:GAGG SNPs, Ce:GAGG MPs, Eu:GAGG particles, (C_38_H_34_P_2_)MnBr_4_ particles, and Cs_3_Cu_2_I_5_ nanocrystals) were sonicated for 30 min and added to the wells with a final concentration of 5 or 50 μg/mL. After a 24-h incubation, bright-field images were captured using a microscope (BZ-X800, Keyence, Osaka, Japan). Due to the microscope’s compatibility with the 96-well plates, we used a 4X objective lens (PlanApo 4X, Keyence) with a numerical aperture (NA) of 0.20. Immedi-ately after imaging, cell viability was measured using the Cell Counting Kit-8 (CCK8, Dojindo, Kumamoto, Japan) according to the manufacturer’s protocol. The data were normalized to the control value without any scintillator particles after subtracting the value from blank wells containing each corresponding scintillator solution.

### 4.4. Viral Production

For AAV production, HEK293 cells were transfected with vector plasmids, including pAAV encoding ChRmine or hrGFP together with pHelper, and pAAV-RC (serotype 9), using the standard calcium phosphate method. After three days, transfected cells were collected and suspended in lysis buffer (150 mM NaCl, 20 mM Tris, pH 8.0). Following four days freeze–thaw cycles, the cell lysate was treated with 250 U/mL benzonase nuclease (Merck) at 37 °C for 10–15 min, with the addition of 1 mM MgCl_2_, and then centrifuged at 1753× *g* for 20 min at 4 °C. AAV was then purified from the supernatant using iodixanol gradient ultracentrifugation. The purified AAV solution was concentrated in PBS via filtration and stored at −80 °C.

### 4.5. Animals

Adult C57BL6/J wild-type mice and DAT-IRES-Cre mice (B6.SJL-*Slc6a3^tm1.1(cre)Bkmn^*/J, The Jackson Laboratory, Bar Harbor, MN, USA) of both sexes were maintained on a 12/12 h light/dark cycle with controlled humidity (40–60%) and temperature (21–26 °C). Mice had free access to food and water. Only 10−16-week-old male DAT-IRES-Cre mice (homozygous or heterozygous mutants) were used for the CPP experiments.

### 4.6. Surgery

Mice were anesthetized with isoflurane (3.0–3.5% for induction, 1.0–1.5% for maintenance) and head-fixed on a stereotactic device using ear bars and a nose clamp. Body temperature was maintained at ~37 °C using a controllable heating pad. An ocular ointment was applied to the eyes to prevent drying. AAV9-CaMKIIa-ChRmine-eYFP-WPRE (original titer: 8.7 × 10^12^ copies/mL, diluted to 1/5), AAV9-Ef1a-DIO-ChRmine-eYFP-WPRE (titer: 2.3 × 10^13^ copies/mL, diluted to 1/10), or AAV9-CMV-DIO-hrGFP (titer: 6.24 × 10^11^ copies/mL) was injected into the hindlimb region of the primary somatosensory cortex (S1) (AP: −0.5 mm; ML: ±1.5 mm; depth: 0.8 and 0.35 mm from the pial surface) of wild-type C57BL6/J mice or the VTA (AP: −3.0–3.3 mm, ML: ±0.5 mm, depth: 4.2 mm) of DAT-IRES-Cre mice through small craniotomies. The injection volume was 400 nL (200 nL at each depth of S1) or 200 nL (VTA) per site. After AAV injection, mice were kept in their home cages for at least three weeks before electrophysiological and behavior experiments. For electrophysiological recordings, SNPs or Eu:GAGG particles (50 mg/mL in Ringer’s solution) were unilaterally or bilaterally injected into S1 (depth: 0.5 mm) or VTA (depth: 4.2 mm) with a volume of 600 nL per site at the coordinates of AAV injection (except for hrGFP-expressing mice, see below). For CPP tests, SNPs were injected bilaterally into a total of four sites of VTA (AP: −3.0–3.3 mm; ML: ±0.5 mm and ±0.2 mm; depth: 4.2 mm). For electrophysiological recording from hrGFP-expressing mice, we used the mice after CPP experiments (Table S2). Before injection, the solution containing the scintillator particles was sonicated for a minimum of 30 min to attenuate the aggregation of particles. For in vivo toxicity tests, we injected scintillator particles (50 mg/mL, 600 nL) into the VTA unless otherwise noted.

Prior to electrophysiological experiments, mice were anesthetized and head-fixed as described above. A small metal screw was implanted over the cerebellum to serve as a reference electrode. A lightweight metal head holder (CF-10, Narishige, Tokyo, Japan) was then implanted over the dorsal skull, aligned parallel to the anterior–posterior and lateral–medial axes, using dental cement. A recording chamber was built with dental cement around the craniotomies. The exposed skull was covered with a thin layer of dental cement, and the chamber was filled with a silicone elastomer to protect the craniotomies and the skull until the recording day.

### 4.7. Histology

For histological analysis, we performed transcardial perfusion with 4% paraformaldehyde (PFA) in phosphate buffer. The brains were removed and incubated in 4% PFA overnight for post-fixation. The fixed brains were then kept in phosphate-buffered saline (PBS) until further processing. Subsequently, they were sectioned into coronal slices on a vibratome (section thickness: 80 μm). For immunostaining analysis, the slices were washed three times with a blocking buffer containing 1% bovine serum albumin (BSA) and 0.25% Triton-X in PBS before being incubated with primary antibodies (anti-Iba1, rabbit monoclonal, 1:500, Fujifilm Wako Pure Chemical Corporation, #019-19741; anti-GFAP, mouse monoclonal, 1:1000, Merck Millipore, Burlington, MA, USA, MAB360) in the blocking buffer overnight at 4 °C. Following incubation, the slices were washed three times with the blocking buffer and were then incubated with secondary antibodies (CF594-conjugated donkey anti-rabbit IgG, 1:1000, Biotium, Fremont, CA, USA, #20152; CF488A-conjugated goat anti-mouse IgG, 1:1000, Biotium, #20018) and DAPI (4’,6-diamidino-2-phenylindole, 2 μM in phosphate buffer), used to stain cellular nuclei, in the blocking buffer for 2–3 h at RT. In some experiments, we only stained slices with DAPI for 25–30 min at RT. The stained samples were mounted using an anti-photobleaching reagent DABCO (1,4-diazabicyclo [2.2.2]octane) and were consistently kept in the dark before observation. Epi-fluorescence imaging was conducted under a fluorescence microscope (BZ-X800, Keyence) using the BZ-X Viewer software (version 01.02.03.02, Keyence). For semi-quantitative analysis of the immunostained slices, we used a 4X objective lens (PlanApo 4X, Keyence) with an NA of 0.20. Images shown in the figures were captured using a 10X objective lens (PlanApo 10X, Keyence) with an NA of 0.45 unless otherwise noted. The fluorescence band-pass filter settings were as follows (in nm): for blue imaging, excitation 395/25, dichroic mirror 425, emission 460/50; for green imaging, excitation 470/40, dichroic mirror 495, emission 525/50; for red imaging, excitation 560/40, dichroic mirror 585, emission 635/75. To minimize photobleaching, we illuminated samples with excitation light only during imaging.

### 4.8. In Vivo Electrophysiology Under an X-Ray Machine

Prior to recording, mice were habituated for head fixation for three days (one session per day), lasting for 15 min in the first session, 30 min in the second session, and 45 min in the third session. Since our X-ray machine (detailed below) produces sound noise during stimulation, we applied broadband white noise at around 80 dB during recording to mask the sound. The mice were habituated to this white noise during the head fixation habituation sessions to minimize startle responses during recordings. On the day of recording, the small craniotomy made for AAV or scintillator injection into S1 or VTA was enlarged to have a diameter of less than 1 mm. Extracellular neuronal firings were recorded in head-fixed mice using a silicon probe (A1 × 32-Poly2-10mm50s-177, NeuroNexus) with 32 recording sites along a single shank, covering 775 mm of cortical depth. The recording chamber was filled with Ringer’s solution. The probe was then slowly lowered (~2 mm/s) to a depth of 0.9–1.1 mm (S1) or 4.6–4.7 mm (VTA) beneath the pial surface. For cortical recordings, all steps during the surgery and probe insertion were performed in conditions minimizing light exposure to prevent unintended over-activation of ChRmine and cortical circuits. Neural data were filtered between 2.5 Hz and 7.6 kHz, amplified using a digital head-stage (RHD2132, Intan Technologies, Los Angeles, CA, USA), and digitized with a sampling frequency of 30 kHz. The digitized neural signal was transferred to an acquisition board (Open Ephys, Atlanta, GA, USA) and stored on the host PC’s internal HDD for offline analysis. Before insertion, the probe was stained by carefully applying DiI (1,1’-dioctadecyl-3,3,3’,3’-tetramethylindocarbocyanine perchlorate) solution (1% in ethanol) with a brush on the backside of the silicon probe to label the recording site. After recordings, the mice were perfused with 4% PFA, and the brain was histologically analyzed to confirm the location of the probe insertion and scintillator particle injection. Since the range of recording electrodes of the silicon probe (775 μm) exceeds the total vertical range of the VTA (~600 μm), we carefully analyzed stained brain slices for the silicon probe trace. We only included neuronal data in our analysis that were confirmed to be within the VTA.

For X-irradiation, we used an X-ray tube (0.6/1.2P18DE-85, Shimadzu, Kyoto, Japan) with an X-ray beam limiting device (R-20, Shimadzu). Before recording, the X-ray tube was preheated with nine 0.1 s pulses of 100 mA tube current with varying tube voltages (three 60 kV, three 90 kV, and three 120 kV). The X-ray beam limiting device was positioned 100 cm above the mouse’s head, center at the recording site. Recording commenced 1 h after the silicon probe was placed at the target to ensure stability. White noise was administered near the mouse’s ears using headphones to mask sound noises during X-irradiation, and 0.5 s X-ray pulses were irradiated during recording at 120 kV tube voltage and varying tube currents. To synchronize X-irradiation with neuronal recordings, we used a photosensor (2151, Newport, Irvine, CA, USA) that detects radioluminescence from bulk Ce:GAGG crystals placed near the mouse within the X-irradiation area. The bulk crystals were covered in aluminum foil, and the radioluminescence was guided through an optical fiber to the photosensor. The photosensor signals were recorded via the acquisition board.

### 4.9. Conditioned Place Preference Test

More than two weeks after the AAV injections, mice were bilaterally injected with SNPs (50 mg/mL in Ringer’s solution) the week before the behavioral tests. The CPP tests were conducted using the X-ray machine employed for electrophysiological recordings. Testing occurred under white LED lighting during the dark period of the 12/12 h light/dark cycle. The mice were placed in their home cage in the experimental room 1 h prior to the experiment. The CPP test chamber had two compartments with different floor textures and visual cues on all walls of each compartment, except for the wall separating the two compartments. Before the CPP test, ChRmine-expressing mice and hrGFP-expressing control mice were habituated to the test chamber for three days (15 min per day) in a free-moving condition, allowing them to explore both chambers through a gated wall. On the first day of the test, mice were placed into the test chamber and allowed to explore freely for 10 min without X-irradiation (pre-test). During each of the following four conditioning days, mice were confined to one of the two compartments for approximately 15 min, then later to the other compartment for another 15 min. During confinement in one of the compartments, the mice received 0.5 s X-irradiation (25 pulses every 30 s, at 20 mGy/s with tube voltage 120 kV and tube current 200 mA). We set a standard distance of 100 cm between the X-ray source and the mouse during locomotion (Figure S3). The total X-ray dose administered during the CPP experiments was estimated to be 1 Gy. Mice exhibited rearing behavior, bringing them closer to the X-ray source, for 20 ± 4% of the time (*n* = 3 mice). However, this behavior only resulted in a negligible increase of 2.6% in the total X-ray dose. On the final day, the mice were placed back into the test chamber and allowed to explore freely for 10 min (post-test). During the pre- and post-tests, the mice were filmed from above. The movies were analyzed offline as below. After completing the CPP tests, the mice were perfused and subjected to further histological analysis.

### 4.10. Data Analysis

To evaluate the neuroinflammatory effect of scintillator particles injected into the brain, we employed a semi-quantitative approach using ImageJ, following a previously described method [20]. Briefly, to minimize variability in fluorescent intensities across samples, which can result from differences in immunostaining performed on different days, we created binary images from the epi-fluorescence images after background subtraction. The background signal was obtained by averaging the fluorescence intensity of three 100 μm × 100 μm squares in the non-injection sites. Three 100 μm × 100 μm squares around the trace of the particle injection site were drawn, and the fraction of pixels in those squares that exceeded the threshold was calculated.

To analyze the cluster size of injected SNPs (Figure S2c), we selected the brain section containing the largest SNP cluster per injection for each mouse and measured the longest horizontal diameter of the SNP cluster. Data from immunostained slices were excluded to avoid potential inaccuracies due to particle washout during the staining process.

For analyzing neuronal firings, spiking activity recorded on silicon probes was detected and sorted into different clusters using Kilosort 2.5 (https://github.com/cortex-lab/KiloSort (accessed on 18 October 2024)). After an automated clustering step, clusters were manually sorted and refined using Phy (https://github.com/cortex-lab/phy (accessed on 18 October 2024)). Only well-isolated single units were included in the dataset. For classifying units as excited, inhibited, or non-modulated during X-irradiation, we compared the firing rates of individual units between the baseline period and during X-irradiation. *p*-values were then adjusted using the Benjamini–Hochberg procedure to control the false discovery rate [43,51].

To quantify the mouse’s place preference in the CPP tests, we tracked the ears and tail of the mice offline using DeepLabCut [20,42,52]. We trained a deep neural network by manually annotating the left and right ears and tail base of mice using 20–80 frames per movie. The center of mass of these three points was used to determine the mice’s location in the chamber. Animal trajectories (Figure 6c) were generated using custom-made MATLAB programs (version: R2021b). The heat maps shown in Figure 6c indicate the probability of the presence of the animal trajectory within each 100 × 100-pixel image. We excluded the area adjacent to the gate from the analysis (Figure 6c). The CPP score was calculated as the difference in time spent in the X-irradiated compartment between the pre-test and post-test, divided by the total time spent in all compartments during both tests, and expressed as a percentage.

All values are expressed as mean ± SD, except for the PSTHs displayed in Figure 3, Figure 4 and Figure 5, where values are presented as mean ± SEM to better visualize the precision of the mean response over time. All statistical tests except for statistical power analysis were performed using GraphPad Prism. Statistical power was calculated using G*power 3.1 [53]. The normality of data distribution was routinely tested using the Shapiro–Wilk test. Two-sample comparisons were analyzed using unpaired or paired *t*-tests for normally distributed samples and Mann–Whitney *U* tests when at least one of the samples was not normally distributed. Tests for two-sample comparison were two-sided. Statistical analyses for multiple comparisons with normal distribution were performed using one-way ANOVA followed by Bonferroni’s test or Dunnett’s multiple comparison test versus the control. Multiple comparisons with at least one non-normally distributed sample group were conducted using the Kruskal–Wallis test, followed by Dunn’s multiple comparisons test unless otherwise noted. Detailed statistical tests are provided in Table S1.

We did not set any specific assumptions about the results prior to the experiments. Sample sizes were therefore chosen based on standard practices in similar studies [20,23,24,36,37,42] and the availability of animals, with the goal of achieving adequate power. While the post-hoc power analysis presented in Table S1 shows that the power is lower for some tests (e.g., power values below 0.1 in certain comparisons), these cases are primarily associated with small effect sizes or subtle differences between groups. Importantly, the key findings remain robust, as the tests with higher effect sizes consistently demonstrate sufficient power (>0.8 in tests with *p* < 0.01). The statis-tical analyses consistently show effect sizes and *p*-values that align with our conclusions, even in cases where the power is reduced. The consistency of the observed effects across multiple experimental conditions (e.g., in vitro cell viability tests and in vivo neuroinflammation tests), as well as across different types of tests (e.g., comparisons of mean firing rates and proportions of cellular categories), supports the overall validity of our conclusions.

## Data Availability

The original contributions presented in the study are included in the article/Supplementary Materials, further inquiries can be directed to the corresponding author.

## Supplementary Materials

The following supporting information can be downloaded at https://www.mdpi.com/article/10.3390/ijms252111365/s1.
