# Supplementary material for: Comparative Validation of Scintillator Materials for X-Ray-Mediated Neuronal Control in the Deep Brain
Source: Int J Mol Sci. 2024 Oct 22;25(21):11365. doi: 10.3390/ijms252111365 (PMC11547033; doi:10.3390/ijms252111365)
Supplement: Supplementary file 1 [file ijms-25-11365-s001.zip › ijms-3191714-supplementary.pdf]

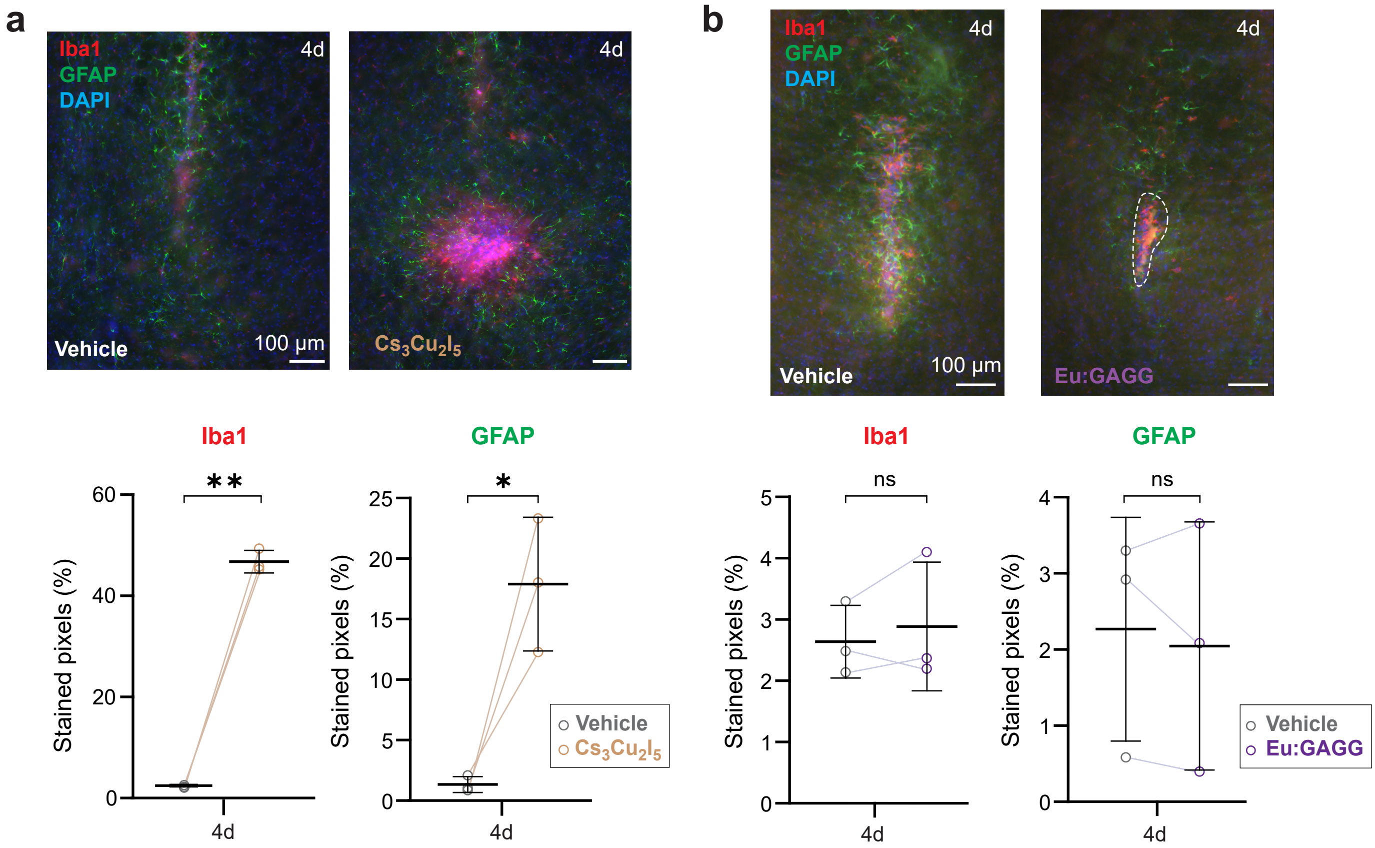


**Figure S1.** In vivo neuroinflammation test for $Cs_3Cu_2I_5$ and Eu:GAGG particles. (**a**) Top, epi-fluorescence images of coronal brain slices showing immunostaining for microglia (Iba1, red) and astrocytes (GFAP, green) at the injection sites of either vehicle (left, 200 nl, Ringer's solution) or $Cs_3Cu_2I_5$ nanocrystals (right, 200 nl, 50 mg/ml). Vehicle and the nanocrystals were injected into different hemispheres of the same mouse, with slices collected 4 days (4d) post-injection. Blue: DAPI. Bottom, semi-quantification of Iba1 (left) and GFAP (right) immunoreactivity expressed as the percentage of stained pixels in a 100 μm × 100 μm square near the injection traces ($n$ = 3 for each). ** $p$ = 0.001, * $p$ = 0.0430, paired $t$-test. (**b**) Same as (**a**), but with injected Eu:GAGG particles (50 mg/ml, 600 nl; dashed outline). $n$ = 3 for each. ns, not significant, paired $t$-test. Open circles represent individual data points. Colored lines connecting open circles indicate individual mice. Black horizontal lines and error bars indicate mean ± SD. Statistical details are shown in Table S1.

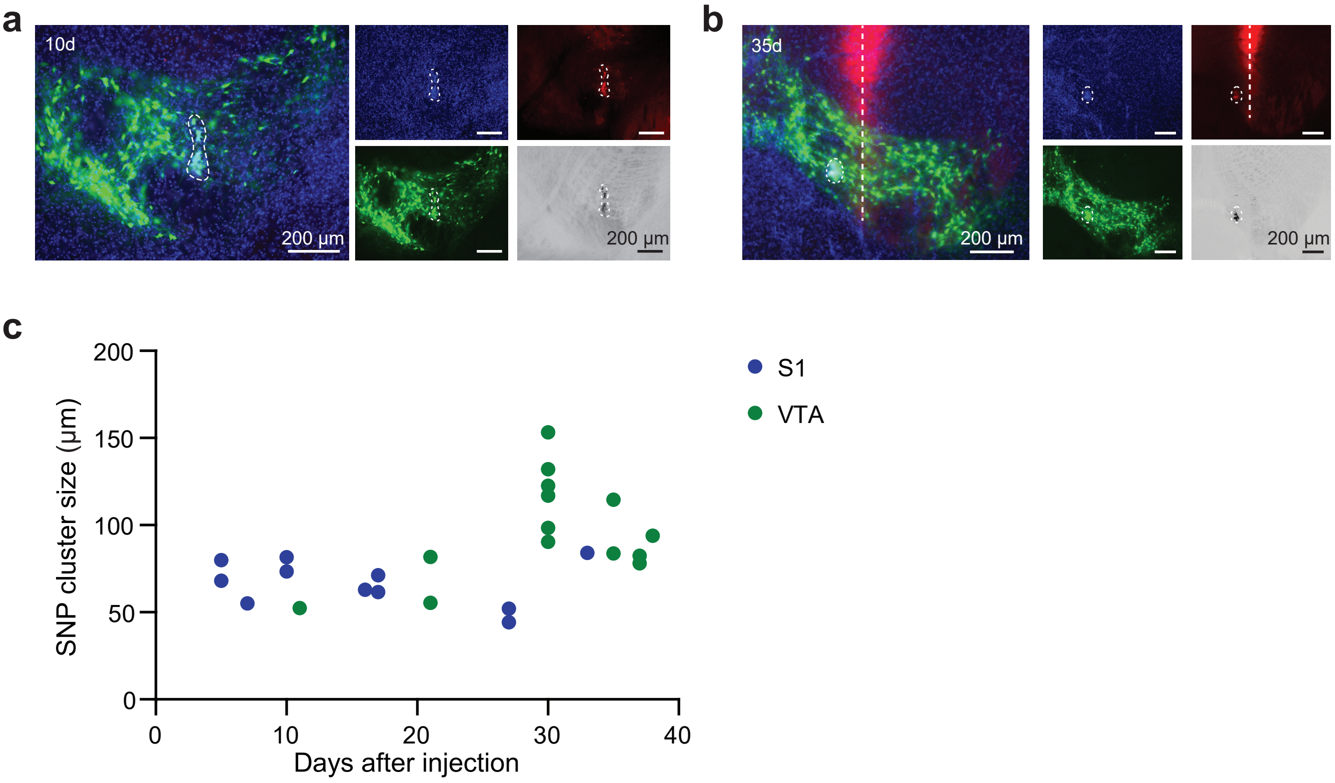


**Figure S2.** Injected SNPs may remain stable at the injection site. (**a**) Left, representative epi-fluorescence image of hrGFP-expressing VTA-DA neurons (green) and injected SNPs (outlined with white dotted lines). The mouse brain was analyzed 10 days (10 d) after SNPs injection. The image is the merged view of red, green, and blue channels. Right, images from individual blue (top left), red (top right), and green (bottom left) channels of the merged image on the left. A bright field image is also shown (bottom right). SNPs were identifiable in the red, green, and blue channels, as well as in the bright field image. (**b**) Same as (**a**) , but with a mouse brain 35 days (35 d) after SNPs injection. This particular example also includes an electrode trace labeled with DiI (visible in the red channel) indicated by the white dotted vertical lines. (**c**) Cluster size of SNPs injected into S1 (blue) and VTA (green) measured at different days after injection. (S1: $n$ = 11 injection sites from 10 mice; VTA: $n$ = 14 injection sites from 8 mice). The cluster size did not significantly changed over time (S1: $r$ = -0.2406, $p$ = 0.4761, Pearson's correlation, two-sided; VTA: $r$ = 0.4039, $p$ = 0.1521; Pearson's correlation, two-sided). Individual data points represent separate injections, including cases where mice used for behavioral experiments received two injections in the same hemisphere. Statistical details are shown in Table S1.

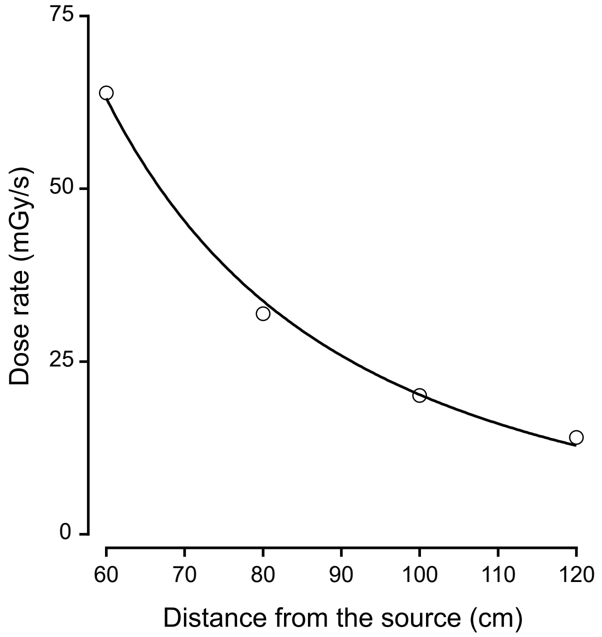


**Figure S3.** X-ray dose rates as a function of distance from the X-ray source. The X-ray dose rates were measured at various distances from the source, with the X-ray tube voltage set to 120 kV and the tube current at 200 mA. The data were fitted to the inverse square law, demonstrating that the dose rate decreases with the square of the distance from the source. For behavioral experiments, we selected a standard distance of 100 cm between the X-ray source and the mouse, which resulted in a consistent dose rate of 20 mGy/s. This relatively long distance minimizes the effect of variations in the mouse's position during the experiments, ensuring relatively consistent X-ray exposure across different trials.

| Figure Number and Data Description | | Normality | Applied Statistical Test | Test Statistics | *P*-value | Effect size | Power |
|---|---|---|---|---|---|---|---|
| **Figure 1b** | Control vs. SNPs, 5 μg/ml | Yes | Dunnett's test (n = 6 vs. 6) | $q = 1.037$ | 0.7499 | $d = –0.7065$ | 0.291 |
| | Control vs. SMPs, 5 μg/ml | Yes | Dunnett's test (n = 6 vs. 6) | $q = 0.6273$ | 0.9544 | $d = –0.4426$ | 0.144 |
| | Control vs. Eu:GAGG, 5 μg/ml | Yes | Dunnett's test (n = 6 vs. 6) | $q = 0.3519$ | 0.9963 | $d = –0.1604$ | 0.0621 |
| | Control vs. $Cs_3Cu_2I_5$, 5 μg/ml | Yes | Dunnett's test (n = 6 vs. 6) | $q = 2.607$ | 0.0559 | $d = 1.404$ | 0.784 |
| | Control vs. $(C_{38}H_{34}P_2)MnBr_4$, 5 μg/ml | Yes | Dunnett's test (n = 6 vs. 6) | $q = 6.407$ | < 0.0001**** | $d = 4.283$ | 1.00 |
| | Control vs. SNPs, 50 μg/ml | Yes | Dunnett's test (n = 6 vs. 6) | $q = 0.7473$ | 0.9118 | $d = –0.4121$ | 0.132 |
| | Control vs. SMPs, 50 μg/ml | Yes | Dunnett's test (n = 6 vs. 6) | $q = 0.6392$ | 0.9509 | $d = 0.3306$ | 0.102 |
| | Control vs. Eu:GAGG, 50 μg/ml | Yes | Dunnett's test (n = 6 vs. 6) | $q = 1.264$ | 0.5939 | $d = 0.9193$ | 0.445 |
| | Control vs. $Cs_3Cu_2I_5$, 50 μg/ml | Yes | Dunnett's test (n = 6 vs. 6) | $q = 8.408$ | < 0.0001**** | $d = 5.163$ | 1.00 |
| | Control vs. $(C_{38}H_{34}P_2)MnBr_4$, 50 μg/ml | Yes | Dunnett's test (n = 6 vs. 6) | $q = 18.46$ | < 0.0001**** | $d = 19.83$ | 1.00 |
| **Figure 1d** | Vehicle vs. SNPs, Iba1, 4d | Yes | Paired *t*-test (n = 3 vs. 3) | $t_2 = 1.350$ | 0.3095 | $d = 0.7794$ | 0.131 |
| | Vehicle vs. SNPs, Iba1, 1w | Yes | Paired *t*-test (n = 3 vs. 3) | $t_2 = 0.5590$ | 0.6324 | $d = 0.3228$ | 0.0644 |
| | Vehicle vs. SNPs, Iba1, 4w | Yes | Paired *t*-test (n = 3 vs. 3) | $t_2 = 0.6208$ | 0.5981 | $d = -0.3584$ | 0.0677 |
| | Vehicle vs. SNPs, GFAP, 4d | Yes | Paired *t*-test (n = 3 vs. 3) | $t_2 = 0.1116$ | 0.9214 | $d = –0.06438$ | 0.0506 |
| | Vehicle vs. SNPs, GFAP, 1w | Yes | Paired *t*-test (n = 3 vs. 3) | $t_2 = 1.549$ | 0.2614 | $d = 0.8945$ | 0.155 |
| | Vehicle vs. SNPs, GFAP, 4w | Yes | Paired *t*-test (n = 3 vs. 3) | $t_2 = 0.9274$ | 0.4516 | $d = –0.5353$ | 0.0890 |
| **Figure 3b** | SNPs+ ChRmine+ vs. SNPs- ChRmine+ | No | Dunn's test (n = 82 vs. 76) | $Z = 2.426$ | 0.0458* | $r_\beta = 0.1930$ | 0.670 |
| | SNPs+ ChRmine+ vs. SNPs+ ChRmine- | No | Dunn's test (n = 82 vs. 80) | $Z = 2.768$ | 0.0169* | $r_\beta = 0.2175$ | 0.805 |
| | SNPs+ ChRmine+ vs. SNPs- ChRmine- | No | Dunn's test (n = 82 vs. 138) | $Z = 3.670$ | 0.0007*** | $r_\beta = 0.2474$ | 0.954 |
| **Figure 3c** | SNPs+ ChRmine+ vs. SNPs- ChRmine+ | - | Chi-square test with Bonferroni's correction | $\chi^2 = 51.32$ | < 0.0001**** | $V = 0.5699$ | 1.00 |
| | SNPs+ ChRmine+ vs. SNPs+ ChRmine- | - | Chi-square test with Bonferroni's correction | $\chi^2 = 47.41$ | < 0.0001**** | $V = 0.5410$ | 1.00 |
| | SNPs+ ChRmine+ vs. SNPs- ChRmine- | - | Chi-square test with Bonferroni's correction | $\chi^2 = 53.54$ | < 0.0001**** | $V = 0.5037$ | 1.00 |
| **Figure 3d** | Proportion of excited neurons w/ different dose rates | No | Kruskal-Wallis test | $H = 15.88$ | 0.0012** | $\eta^2 = 0.8050$ | 0.999 |
| **Figure 4a** | Eu:GAGG+ ChRmine+ vs. Eu:GAGG- ChRmine+ | No | Dunn's test (n = 101 vs. 76) | $Z = 0.6754$ | 0.9989 | $r_\beta = 0.05077$ | 0.100 |
| | Eu:GAGG+ ChRmine+ vs. Eu:GAGG- ChRmine- | No | Dunn's test (n = 101 vs. 138) | $Z = 0.8975$ | 0.7389 | $r_\beta = 0.05805$ | 0.0716 |
| **Figure 4b** | Eu:GAGG+ ChRmine+ vs. Eu:GAGG- ChRmine+ | - | Chi-square test with Bonferroni's correction | $\chi^2 = 6.322$ | 0.0848 | $V = 0.1773$ | 0.550 |
| | Eu:GAGG+ ChRmine+ vs. Eu:GAGG- ChRmine- | - | Chi-square test with Bonferroni's correction | $\chi^2 = 5.961$ | 0.1856 | $V = 0.1438$ | 0.499 |
| **Figure 5d** | SNPs+ ChRmine+ vs. SNPs+ ChRmine- (hrGFP+) | No | Dunn's test (n = 60 vs. 78) | $Z = 2.927$ | 0.0068** | $r_\beta = 0.2492$ | 0.829 |
| | SNPs+ ChRmine+ vs. SNPs- ChRmine- | No | Dunn's test (n = 60 vs. 98) | $Z = 4.525$ | < 0.0001**** | $r_\beta = 0.3560$ | 0.994 |
| **Figure 5e** | SNPs+ ChRmine+ vs. SNPs+ ChRmine- (hrGFP+) | - | Chi-square test with Bonferroni's correction | $\chi^2 = 19.00$ | 0.000150*** | $V = 0.3711$ | 0.981 |
| | SNPs+ ChRmine+ vs. SNPs- ChRmine- | - | Chi-square test with Bonferroni's correction | $\chi^2 = 22.54$ | < 0.0001**** | $V = 0.3777$ | 0.993 |
| **Figure 6d** | Day 0 vs. Day 5, ChRmine | Yes | Paired *t*-test (n = 7 vs. 7) | $t_6 = 2.705$ | 0.0353* | $d = 1.022$ | 0.618 |
| | Day 0 vs. Day 5, hrGFP | Yes | Paired *t*-test (n = 7 vs. 7) | $t_6 = 1.774$ | 0.1264 | $d = –0.6711$ | 0.321 |
| **Figure 6e** | ChRmine vs. hrGFP | Yes | Unpaired *t*-test (n = 7 vs. 7) | $t_{12} = 0.5160$ | 0.6153 | $d = –0.2754$ | 0.0763 |
| **Figure 6f** | ChRmine vs. hrGFP | No | Mann-Whitney *U* test (n = 7 vs. 7) | $U = 3$ | 0.0041** | $r_\beta = 0.8776$ | 1.00 |
| **Figure S1c** | Vehicle vs. $Cs_3Cu_2I_5$, Iba1 | Yes | Paired *t*-test (n = 3 vs. 3) | $t_2 = 31.31$ | 0.0010** | $d = 18.08$ | 1.00 |
| | Vehicle vs. $Cs_3Cu_2I_5$, GFAP | Yes | Paired *t*-test (n = 3 vs. 3) | $t_2 = 4.664$ | 0.0430* | $d = 2.694$ | 0.671 |
| **Figure S1d** | Vehicle vs. Eu:GAGG, Iba1 | Yes | Paired *t*-test (n = 3 vs. 3) | $t_2 = 0.7869$ | 0.5138 | $d = 0.4543$ | 0.0782 |
| | Vehicle vs. Eu:GAGG, GFAP | Yes | Paired *t*-test (n = 3 vs. 3) | $t_2 = 0.6451$ | 0.5850 | $d = –0.3724$ | 0.0691 |
| **Figure S2c** | Cluster size distribution for S1 injections | Yes | Pearson correlation (n = 11) | - | 0.4761 | $r = -0.2406$ | 0.108 |
| | Cluster size distribution for VTA injections | Yes | Pearson correlation (n = 14) | - | 0.1521 | $r = 0.4039$ | 0.308 |

**Table S1.** Statistical details. For effect size measures, *d* represents Cohen's *d*; $r_\beta$ denotes rank-biserial correlation; $\eta^2$ is Kruskal-Wallis effect size; *V* indicates Cramér's *V*; and *r* represents Pearson correlation coefficient.

| | | Date (DD.MM.YYYY)<br>Days before recording | | |
|---|---|---|---|---|
| Mouse ID | Hemisphere | AAV Injection | Particle Injection | Recording |
| | | **S1 Recording** | | |
| **SNPs+ ChRmine+ (*n* = 7 hemispheres from 5 mice)** | | | | |
| MH045 | Right | 30.06.2023<br>27 | 11.07.2023<br>16 | 27.07.2023 |
| | Left | 30.06.2023<br>28 | 11.07.2023<br>17 | 28.07.2023 |
| MH058 | Left | 11.09.2023<br>24 | 29.09.2023<br>6 | 05.10.2023 |
| | Right | 11.09.2023<br>35 | 06.10.2023<br>10 | 16.10.2023 |
| MH066 | Left | 13.10.2023<br>34 | 09.11.2023<br>7 | 16.11.2023 |
| MH110 | Left | 02.05.2024<br>35 | 31.05.2024<br>6 | 06.06.2024 |
| MH111 | Right | 02.05.2024<br>42 | 07.06.2024<br>6 | 13.06.2024 |
| **SNPs- ChRmine+ (*n* = 8 hemispheres from 6 mice)** | | | | |
| MH050 | Left | 01.08.2023<br>23 | - | 24.08.2023 |
| MH053 | Right | 21.08.2023<br>31 | - | 21.09.2023 |
| MH063 | Left | 11.09.2023<br>38 | - | 19.10.2023 |
| | Right | 11.09.2023<br>42 | - | 23.10.2023 |
| MH064 | Right | 19.09.2023<br>41 | - | 30.10.2023 |
| MH097 | Right | 01.03.2024<br>21 | - | 22.03.2024 |
| | Left | 01.03.2024<br>21 | - | 22.03.2024 |
| MH098 | Left | 01.03.2024<br>28 | - | 29.3.2024 |
| **SNPs+ ChRmine– (*n* = 4 hemispheres from 2 mice)** | | | | |
| MH068 | Right | - | 15.11.2023<br>15 | 30.11.2023 |
| | Left | - | 15.11.2023<br>16 | 01.12.2023 |
| MH069 | Left | - | 04.12.2023<br>7 | 11.12.2023 |
| | Right | - | 04.12.2023<br>7 | 11.12.2023 |
| **EuGAGG+ ChRmine+ (*n* = 5 hemispheres in 3 mice)** | | | | |
| MH096 | Left | 07.03.2024<br>35 | 07.04.2024<br>4 | 11.04.2024 |
| MH099 | Left | 01.04.2024<br>21 | 17.04.2024<br>5 | 22.04.2024 |
| | Right | 01.04.2024<br>24 | 19.04.2024<br>6 | 25.04.2024 |
| MH100 | Left | 03.04.2024<br>22 | 17.04.2024<br>8 | 25.04.2024 |

| | | Date (DD.MM.YYYY)<br>Days before recording | | |
|---|---|---|---|---|
| Mouse ID | Hemisphere | AAV Injection | Particle Injection | Recording |
| | | **VTA Recording** | | |
| **SNPs+ ChRmine+ (*n* = 5 hemispheres from 5 mice)** | | | | |
| MH059 | Right | 08.08.2023<br>23 | 25.08.2023<br>6 | 31.08.2023 |
| MH061 | Left | 30.08.2023<br>21 | 11.09.2023<br>9 | 20.09.2023 |
| MH070 | Right | 12.09.2023<br>30 | 26.09.2023<br>16 | 12.10.2023 |
| MH071 | Left | 12.09.2023<br>44 | 06.10.2023<br>20 | 26.10.2023 |
| MH117 | Right | 26.04.2024<br>38 | 07.05.2024<br>27 | 03.06.2024 |
| **SNPs+ ChRmine– (hrGFP+) (*n* = 4 hemispheres from 3 mice)** | | | | |
| MH124 | Right | 04.06.2024<br>58 | 25.06.2024<br>37 | 01.08.2024 |
| MH133 | Right | 14.06.2024<br>53 | 02.07.2024<br>35 | 06.08.2024 |
| | Left | 14.06.2024<br>55 | 02.07.2024<br>37 | 08.08.2024 |
| MH134 | Left | 14.06.2024<br>55 | 02.07.2024<br>37 | 08.08.2024 |
| | | **Conditioned Place Preference test** | | |
| **SNPs+ ChRmine+ (*n* = 7 mice)** | | | | **CPP d0** |
| MH113 | Both | 09.04.2024<br>34 | 08.05.2024<br>5 | 13.05.2024 |
| MH114 | Both | 09.04.2024<br>34 | 08.05.2024<br>5 | 13.05.2024 |
| MH116 | Both | 26.04.2024<br>24 | 14.05.2024<br>6 | 20.05.2024 |
| MH119 | Both | 06.05.2024<br>21 | 21.05.2024<br>6 | 27.05.2024 |
| MH120 | Both | 06.05.2024<br>21 | 21.05.2024<br>6 | 27.05.2024 |
| MH125 | Both | 04.06.2024<br>27 | 25.06.2024<br>6 | 01.07.2024 |
| MH126 | Both | 04.06.2024<br>27 | 25.06.2024<br>6 | 01.07.2024 |
| **SNPs+ ChRmine– (hrGFP+) (*n* = 7 mice)** | | | | |
| MH121 | Both | 16.05.2024<br>39 | 19.06.2024<br>5 | 24.06.2024 |
| MH123 | Both | 16.05.2024<br>39 | 19.06.2024<br>5 | 24.06.2024 |
| MH124 | Both | 04.06.2024<br>27 | 25.06.2024<br>6 | 01.07.2024 |
| MH133 | Both | 14.06.2024<br>24 | 02.07.2024<br>6 | 08.07.2024 |
| MH134 | Both | 14.06.2024<br>24 | 02.07.2024<br>6 | 08.07.2024 |
| MH135 | Both | 24.06.2024<br>21 | 09.07.2024<br>6 | 15.07.2024 |
| MH136 | Both | 24.06.2024<br>21 | 09.07.2024<br>6 | 15.07.2024 |

**Table S2.** Detailed experimental timeline of individual mice.
